# Selected Metal Matrix Metalloproteinases and Tissue Inhibitors of Metalloproteinases as Potential Biomarkers for Tubulointerstitial Fibrosis in Children with Unilateral Hydronephrosis

**DOI:** 10.1155/2020/9520309

**Published:** 2020-06-29

**Authors:** Beata Bieniaś, Przemysław Sikora

**Affiliations:** Department of Pediatric Nephrology Medical University of Lublin, Gębali 6, 20-093 Lublin, Poland

## Abstract

Renal tubulointerstitial fibrosis caused by congenital ureteropelvic junction obstruction (UPJO) may lead to the development of obstructive nephropathy (ON) and the impairment of kidney function. Hence, the identification of early biomarkers of this condition might be of assistance in therapeutic decisions. This study evaluates serum and urinary metalloproteinases MMP-1, MMP-2, and MMP-9 and tissue inhibitors of metalloproteinases TIMP-1 and TIMP-2 as potential biomarkers of ON in children with congenital unilateral hydronephrosis (HN) caused by UPJO. Forty-five (45) children with congenital HN of different grades of severity and twenty-one (21) healthy controls were enrolled in the study. Urinary and serum concentrations of MMP-1, MMP-2, MMP-9, TIMP-1 and TIMP-2 were measured using specific ELISA kits. The urinary excretions were expressed as biomarker/creatinine (Cr) ratios. To evaluate the extracellular matrix remodelling process activity, the serum and urinary MMP-1, -2, -9/TIMP-1, -2 ratios were also calculated. In comparison with the controls, patients with HN, independent of the grade, showed significantly increased median serum MMP-9, TIMP-1, and TIMP-2, median urinary MMP-9/Cr, and TIMP-2/Cr ratios. Lower median values of serum MMP-2/TIMP-1, MMP-9/TIMP-1 in patients with HN were also revealed. Additionally, higher urinary MMP-2/Cr, lower urinary MMP-2/TIMP-2, and lower serum MMP-9/TIMP-2 ratios were observed in patients with HN grades 3 and 4. Patients with ON diagnosed by renal scintigraphy had a significantly higher median serum MMP-9 concentration and lower median serum MMP-9/TIMP-1, -2 ratios in comparison with those without this condition. Patients with nonglomerular proteinuria had a significantly higher median serum TIMP-1 concentration, a higher median urinary TIMP-2/Cr ratio, and a lower serum MMP-9/TIMP-1 ratio compared to those without this symptom. The relationship between the measured biomarkers and the relative function of the obstructed kidney showed no correlations. The ROC curve analysis showed a promising diagnostic profile for the detection of ON for serum MMP-9 and the serum MMP-9/TIMP-1 and MMP-9/TIMP-2 ratios. In conclusion, the results of this study suggest that patients with HN, particularly with grades 3 and 4, are at higher risk of renal tubulointerstitial fibrosis. The noninvasive markers of this condition considered are urinary MMP-2/Cr and MMP-9/Cr, serum MMP-9, serum and urinary MMP-2, MMP-9/TIMP-1, -2. Additionally, serum MMP-9 and MMP-9/TIMP-1, -2 may become promising markers of ON.

## 1. Introduction

Obstructive nephropathy (ON) caused by congenital ureteropelvic junction obstruction (UPJO) is progressive tubulointerstitial fibrosis leading to glomerular sclerosis and the impairment of kidney function [1, 2]. The pathogenesis of renal fibrosis is complex. The final stages of its development are related to an imbalance between the formation and degradation of the extracellular matrix (ECM).

Multiple factors such as the grade of hydronephrosis, urinary tract infections, and individual considerations are the sole determinants of the mechanism of renal fibrosis and its initiation and progression. Therefore, identification of early biomarkers of this condition might help in better stratification of patients with UPJO and facilitate therapeutic decisions.

In the recent years, matrix metalloproteinases (MMPs), which are proteolytic enzymes that degrade matrix proteins [3] and their tissue inhibitors (TIMPs), have been studied as a potential marker of renal fibrosis.

MMPs are a large family of zinc-containing matrix degrading enzymes including mainly collagenases and stromelysins [4–6]. Recent studies show that MMPs may be implicated in the initiation and progression of kidney fibrosis and the development of chronic kidney disease (CKD) [4, 7–9]. MMP-1, MMP-2, and MMP-9 and TIMP-1 and TIMP-2 contribute to the ECM remodelling processes within the renal interstitium [10, 11].

The gelatinases MMP-2 and MMP-9 cleave denatured collagen, type IV collagen, and laminin [12] with both expressed in different renal structures, including the glomeruli, the proximal and distal tubules, and the collecting ducts [12–17]. They are also pivotal to the recruitment and chemotaxis of inflammatory cells [18]. MMP-2 is also the potential activator of MMP-1 and MMP-9 by cleaving their prodomains [19]. It was observed that MMP-9 contributes to the pathogenesis of renal fibrosis through macrophage recruitment and osteopontin cleavage [20]. Similar to MMP-2, it may induce tubular cell epithelial-mesenchymal transition [20, 21]. In addition, MMP-2 can promote ECM production and accumulation [22]. There is also a positive correlation between the urinary excretions of MMP-2 and MMP-9 and transforming growth factor beta (TGF-beta), confirming their profibrotic action [23]. MMP-1 is interstitial collagenase, which degrades the native collagen and is hypothesised to be an antifibrotic enzyme [24, 25]. However, its role in kidney diseases remains incompletely understood.

“*In vitro*” and animal studies show a higher activity of MMPs and TIMPs in the process of kidney fibrosis [7, 24, 25]. Several clinical studies demonstrated increased serum levels and/or urinary excretions of these MMPs and their inhibitors TIMP-1 and TIMP-2 in patients with CKD making them promising markers for this condition [23, 26]. However, to the best of our knowledge, there are few reports on the evaluation of MMPs and TIMPs in HN.

## 2. Purpose of the Study

The purpose of this study is to evaluate the serum and urinary metalloproteinases MMP-1, MMP-2, and MMP-9 and the tissue inhibitors of metalloproteinases TIMP-1 and TIMP-2 as potential biomarkers of ON in children with congenital unilateral hydronephrosis (HN). These parameters were evaluated in relation to the severity of HN, the presence of signs of ON in renal scintigraphy, the relative function of an obstructed kidney, and the presence of proteinuria.

## 3. Patients

Table 1 shows the baseline characteristics of patients and controls. The study comprised 45 children (31 boys and 14 girls) aged 2–17 years (median, 11.0 years) with congenital unilateral HN secondary to UPJO, diagnosed and treated in our department. Using the Onen HN ultrasound grading system [27], HN was classified as follows: stage 1: solitary dilatation of the renal pelvis; stage 2: the same as stage 1 including dilatation of the renal calices; stage 3: the same as stage 2 including <1/2 (mild-to-moderate) renal parenchymal loss; and finally, stage 4: the same as stage 3 plus >1/2 (severe) renal parenchymal loss (cyst-like kidney with no visually significant renal parenchyma). Accordingly, the patients were divided into three subgroups: A, B, and C. Of the 45 children, 25 (55.6%) with HN grades 3 and 4 were included in group A, with 11 (24.4%) with HN grade 2 in group B and 9 (20%) with HN grade 1 in group C.

Dynamic renal scintigraphy using Technetium-99m-L ethylenedicysteine was performed in all patients to determine signs of ON defined as renal parenchymal defects with decreased relative function of the obstructed kidney. Accordingly, ON was revealed in 28/45 (62.2%) patients. There were 21 patients from group A and 7 from group B. The split function of the obstructed kidney compared to the normal kidney ranged from 15 to 85% and 35 to 65%.

Pathological, nonglomerular proteinuria, as an indication of tubular injury (urinary protein/creatinine ratio: >0.21 mg/mg, median: 0.24 mg/mg, range: 0.21–0.4 mg/mg), was revealed in 10 patients. All were from group A, and 7 had signs of ON.

All patients had a normal estimated glomerular filtration rate (eGFR) > 90 ml/min/1.73 m^2^ calculated by the Schwartz formula [28]. Twenty-one age- and sex-matched healthy children were used as controls. They were referred to our outpatient clinic with suspicion of voiding disorders that were subsequently not confirmed.

To evaluate the designed laboratory parameters, the midstream first-morning urine and serum samples were simultaneously collected from each study participant.

MMP-1, MMP-2, and MMP-9 and TIMP-1 and TIMP-2 concentrations were measured using specific enzyme-linked immunosorbent assay (ELISA) kits following the manufacturer's instructions (R&D Systems).

Serum and urinary creatinine concentrations were determined by Jaffe's test. Standard laboratory techniques were used to assess the magnitude of proteinuria.

Urinary excretions of MMP-1, MMP-2, MMP-3, TIMP-1, TIMP-2, and protein were expressed as ratios over creatinine concentration (pg/mg Cr and mg/mg Cr, respectively).

In addition, to evaluate the ECM remodelling process activity, the serum and urinary MMP-1, -2, -9/TIMP-1, -2 ratios (pg/pg) were also calculated.

The statistical analysis was performed using STATISTICA 12.5. The differences between the groups were assessed using a nonparametric Mann-Whitney *U* test, while correlation coefficients were calculated using the Spearman test. A *p* value ≤ 0.05 was considered significant. To determinate the diagnostic utility of the evaluated biomarkers, the receiver operating characteristic (ROC) curves were analysed.

## 4. Ethics Statement

The study was approved by the ethics committee of the Medical University of Lublin.

Informed consent was obtained from all parents or legal representatives, as well as, according to Polish regulations from patients > 16 years of age.

## 5. Results

Tables 2 and 3 show the results of the assessed biomarkers in the study groups compared to the controls. In group A, median serum concentrations of MMP-9, TIMP-1, TIMP-2, and median urinary MMP-2, -9/Cr and TIMP-1, -2/Cr ratios were significantly higher than those in the controls (*p* < 0.05). In comparison with the controls, group A showed significantly lower median values of serum MMP-2/TIMP-1, MMP-9/TIMP-1, -2, and urinary MMP-2/TIMP-1, -2 ratios (*p* < 0.05). In comparison with the controls, patients from group B showed significantly higher median serum MMP-9, TIMP-1, TIMP-2 concentrations, lower median serum MMP-2/TIMP-1, MMP-9/TIMP-1, -2 ratios, and higher median urinary MMP-9/Cr and TIMP-2/Cr ratios (*p* < 0.05). In group C, significantly higher median serum MMP-9, TIMP-1, TIMP-2 concentrations, lower median serum MMP-2, -9/TIMP-1 ratios, and higher median urinary MMP-9/Cr and TIMP-2/Cr ratios in comparison with the controls were observed (*p* < 0.05).

**Figure 1:**
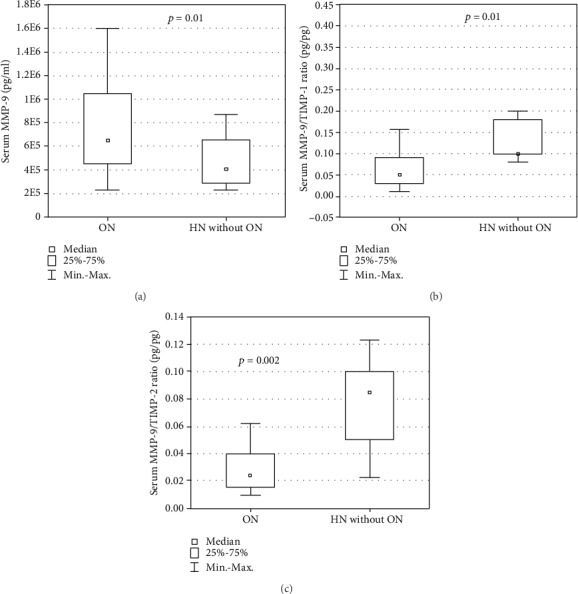
Serum concentration of (a) MMP-9 and serum ratios of (b) MMP-9/TIMP-1 and (c) MMP-9/TIMP-2 in patients with obstructive nephropathy (ON) and hydronephrosis without obstructive nephropathy (HN without ON).

**Figure 2:**
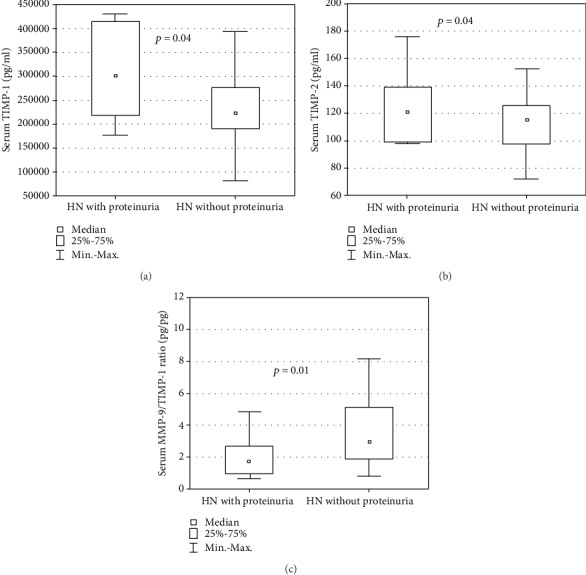
Serum concentrations of (a) TIMP-1 and (b) TIMP-2 and (c) serum MMP-9/TIMP-1 ratio in hydronephrotic patients with and without proteinuria.

Patients with ON had significantly higher median serum MMP-9 concentration (Figure 1) and significantly lower median serum MMP-9/TIMP-1, -2 ratios in comparison with those without this diagnosis (*p* = 0.03, *p* = 0.01, and *p* = 0.002, respectively). In patients with nonglomerular proteinuria, a significantly higher median serum TIMP-1 concentration, a higher median urinary TIMP-2/Cr ratio, and a significantly lower serum MMP-9/TIMP-1 ratio compared to those without this symptom were revealed (*p* < 0.05) (Figure 2). No correlations between the measured biomarkers and the relative function of the obstructed kidney were observed.

**Figure 3:**
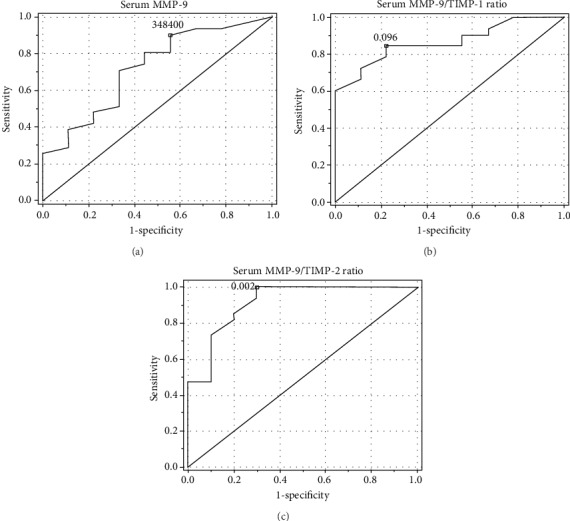
ROC analysis for the (a) serum MMP-9, (b) serum MMP-9/TIMP-1, and (a) serum MMP-9/TIMP-2 ratios in the detection of obstructive nephropathy (patients with obstructive nephropathy (ON) vs. patients with hydronephrosis without obstructive nephropathy (HN without ON)).

The ROC curve analysis showed a promising diagnostic profile for the detection of ON for serum MMP-9 (area under the curve (AUC) of 0.722, optimal cut-off value of 526200 pg/ml with a sensitivity and specificity of 71 and 66.7%, respectively) and also for serum MMP-9/TIMP-1 and MMP-9/TIMP-2 ratios (AUCs of 0.758 and 0.697, optimal cut-off values of 2.961 and 3.838 pg/pg with sensitivities of 58.1 and 83.9% and specificities of 88.9 and 55.6%, respectively) (Figure 3).

## 6. Discussion

Chronic HN may lead to the development of progressive kidney fibrosis determined as ON and the impairment of renal function [29]. Therefore, identification of biomarkers of tubulointerstitial injury would be useful in therapeutic decisions and evaluation of ON progression. Among potential candidates are selected MMPs and TIMPs which contribute to the fibrotic process within the renal tissue [7, 30–32]. However, the amount of relevant clinical data is limited.

The role of MMP-2 in renal fibrosis was shown in several experimental studies [11, 18, 19]. Accordingly, rabbits with experimentally induced UPJO showed higher expression of MMP-2 in the renal cortex [33]. Furthermore, Tveitarås et al. [34] found that MMP-2 knockout and heterozygote mice are protected from kidney fibrosis despite the presence of coexisting UPJO. In a few clinical studies, increased urinary MMP-2 excretion was observed at an early stage of chronic nephropathy [35–38]. Other studies reported patients with CKD with associated progressive kidney fibrosis accompanied by increased serum MMP-2 concentrations [21, 39–44]. In addition, increased serum MMP-2 levels were observed in patients with interstitial fibrosis/tubular atrophy in renal allograft [38]. Our study confirmed an increased release of MMP-2 in chronic uropathy. Patients with HN grades three and four were characterised by higher concentrations of urinary MMP-2 and lower serum and urinary MMP-2/TIMP-1, -2 ratios in comparison with the controls. The latter was also found in patients with mild stages of HN. These results may indicate an association of HN severity, accumulation of ECM in the renal parenchyma, and the development of tubulointerstitial fibrosis. It suggests that serum and urinary MMP-2 could serve as clinical markers of ON. The contribution of MMP-9 in renal fibrosis was evaluated more extensively than MMP-2. Several reports showed increased serum and urinary MMP-9 activity in patients with CKD [40–42]. Abedi and Mohammadjafari [45] postulated that urinary MMP-9 and TIMP-1 may be markers of renal scarring in children with urinary tract infections. Other authors [46] suggested the usefulness of the measurement of urinary MMP-9 excretion and the urinary MMP-9/TIMP-1 ratio for the prediction of vesicoureteral reflux in neonates with antenatal HN. Tian et al. [47] reported that urinary MMP-9 and TIMP-1 may be noninvasive biomarkers in children with UPJO. On the contrary, the data published by Reis et al. [48] showed that higher expression of MMP-9 is a marker for a good surgical outcome in children with UPJO. The authors believe that activation of MMP-9 can reflect increased degradation processes in the ECM. The results of our study suggest increased synthesis and release of MMP-9 in patients with UPJO and the development of renal fibrosis. Children with HN were characterised by significantly increased serum and urinary concentrations of MMP-9. Patients with HN grades two, three, and four also had lower serum MMP-9/TIMP-1, -2 ratios in comparison with the controls. Moreover, in patients with ON, increased serum MMP-9 concentration and decreased serum MMP-9/TIMP-1, -2 ratios were observed. In addition, decreased serum MMP-9/TIMP-1 ratio was also found in patients with HN and nonglomerular proteinuria. Finally, the promising diagnostic profiles of serum MMP-9 and serum MMP-9/TIMP-1, -2 ratios for the detection of ON were confirmed in the ROC curve analysis.

There is only a little data concerning the role of MMP-1 in renal fibrosis. Hirt-Minkowski et al. [49] revealed a significant positive correlation between serum and urinary concentrations of MMP-1 and TIMP-1 and advanced or even initial interstitial fibrosis in renal allograft. In contrast, the data in this study did not show differences in serum and urinary MMP-1 between the groups of patients with HN, with or without ON, and the controls. This may suggest different pathways of fibrosis between native and transplanted kidneys. Therefore, MMP-1 seems not to be a suitable biomarker of CKD caused by ON.

Some experimental studies found higher expression of TIMPs, particularly TIMP-1, in the renal tissue in the course of various kidney diseases [32, 50]. This was confirmed in patients with CKD of different aetiology [23, 51]. It was found that increased urinary excretion of TIMP-1 was observed particularly in patients with UPJO [47] and vesicoureteral reflux [46]. This parameter was also proposed as a predictor of renal scarring [45]. In our study, patients with HN showed higher serum concentrations of TIMP-1 and TIMP-2 and urinary excretions of TIMP-2 in comparison with the controls. Significantly higher excretions of urinary TIMP-1 were found only in children with HN grades three and four. Moreover, in patients with nonglomerular proteinuria, significantly higher serum TIMP-1, higher urinary TIMP-2, and lower serum MMP-9/TIMP-1 ratio compared to those without this symptom were detected. This may confirm the increased ECM remodelling inhibition processes in patients with HN and the higher risk of the development of tubulointerstitial fibrosis.

This study showed that in patients with HN, independent of the grade, the process of renal tubulointerstitial fibrosis is activated. The development or inhibition of this condition is probably dependent on multiple factors, such as the progression of hydronephrosis, urinary tract infections, and also individual considerations. Monitoring of urinary and serum MMP-2, -9 and MMP-2, -9/TIMP-1, -2 ratios may become useful in determining the progression of renal fibrosis and better stratification of patients with ON.

The main limitation of this preliminary study is a relatively small number of patients. Further investigations in patients with HN are required to confirm the utility of serum and urinary MMPs and TIMPs in the diagnosis of ON.

## 7. Conclusions

In conclusion, the results of our study suggest that patients with HN, particularly grades three and four, are at higher risk of renal tubulointerstitial fibrosis. Urinary MMP-2/Cr and MMP-9/Cr, serum MMP-9, and serum and urinary MMP-2, -9/TIMP-1, -2 may be considered as noninvasive markers of this condition. Additionally, serum MMP-9 and MMP-9/TIMP-1, -2 may become promising markers of ON. Therefore, the assessment of these parameters could be useful for follow-up of patients with HN caused by UPJO.

## Figures and Tables

**Table 1:** Characteristics of study and control groups.

Parameter	Study group median (range) and number of patients	Control group
Number of patients	45	21
Gender (male/female)	31/14	16/5
Age (years)	11.0 (2-17)	12.3 (3-17)
GFR (ml/min/1.73 m^2^)	126.3 (97-162)	139 (102-145)
Number of patients in groups A/B/C	25/11/9	—
Number of patients with obstructive nephropathy	28/45 (62.2%)	—
Number of patients with proteinuria	10/45 (22.2%)	—
Protein/creatinine ratio (mg/mg)	0.24 (0.21-0.4)	0.09 (0-0.15)

**Table 2:** The results of serum concentrations of MMPs and TIMPs in study A, B, and C and control groups.

Variable	Group	*N*	Median	Range	Statistical analysis
MMP-1 (pg/ml)	A	25	297.5	410.0-1116.0	A vs. controls *p* = 0.3
	B	11	365.3	800.0-604.5	B vs. controls *p* = 0.2
	C	9	440.1	150.0-930.0	C vs. controls *p* = 0.3
	Controls	21	326.1	209.1-607.1	

MMP-2 (pg/ml)	A	25	416.3 × 10^3^	216.2 × 10^3^‐674.5 × 10^3^	A vs. controls *p* = 0.06
	B	11	435.9 × 10^3^	313.0 × 10^3^‐635.6 × 10^3^	B vs. controls *p* = 0.6
	C	9	416.2 × 10^3^	341.0 × 10^3^‐569.7 × 10^3^	C vs. controls *p* = 0.23
	Controls	21	374.6 × 10^3^	307.2 × 10^3^‐726.2 × 10^3^	

MMP-9 (pg/ml)	A	25	59.1 × 10^3^	22.7 × 10^3^‐159.8 × 10^3^	A vs. controls *p* = 0.0001
	B	11	54.2 × 10^3^	25.1 × 10^3^‐79.3 × 10^3^	B vs. controls *p* = 0.0002
	C	9	52.5 × 10^3^	10.8 × 10^3^‐94.7 × 10^3^	C vs. controls *p* = 0.001
	Controls	21	1.51 × 10^3^	0.90 × 10^3^‐2.2 × 10^3^	

TIMP-1 (pg/ml)	A	25	71.7 × 10^3^	8.20 × 10^3^ − 99.4 × 10^3^	A vs. controls *p* = 0.0001
	B	11	83.0 × 10^3^	19.5 × 10^3^ − 105.1 × 10^3^	B vs. controls *p* = 0.0008
	C	9	75.7 × 10^3^	14.4 × 10^3^ − 98.2 × 10^3^	C vs. controls *p* = 0.001
	Controls	21	13.3 × 10^3^	6.50 × 10^3^ − 18.3 × 10^3^	

TIMP-2 (pg/ml)	A	25	101.5 × 10^3^	7.20 × 10^3^ − 120.6 × 10^3^	A vs. controls *p* = 0.04
	B	11	100.4 × 10^3^	3.86 × 10^3^ − 122.4 × 10^3^	B vs. controls *p* = 0.04
	C	9	100.1 × 10^3^	3.15 × 10^3^ − 120.1 × 10^3^	C vs. controls *p* = 0.04
	Controls	21	90.7 × 10^3^	24.5 × 10^3^ − 173.5 × 10^3^	

MMP-1/TIMP-1	A	25	0.13	0.02-0.2	A vs. controls *p* = 0.5
	B	11	0.14	0.02-0.26	B vs. controls *p* = 0.3
	C	9	0.13	0.04-0.29	C vs. controls *p* = 0.3
	Controls	21	0.1	0.03-0.35	

MMP-2/TIMP-1	A	25	1.6	0.6-2.9	A vs. controls *p* = 0.01
	B	11	1.7	1.1-3.1	B vs. controls *p* = 0.002
	C	9	1.6	1.2-2.0	C vs. controls *p* = 0.001
	Controls	21	2.9	2.4-3.4	

MMP-9/TIMP-1	A	25	0.03	0.01-0.1	A vs. controls *p* = 0.03
	B	11	0.04	0.01-0.1	B vs. controls *p* = 0.04
	C	9	0.15	0.1-2.1	C vs. controls *p* = 0.04
	Controls	21	0.1	0.1-0.2	

MMP-1/TIMP-2	A	25	0.09	0.04-0.2	A vs. controls *p* = 0.4
	B	11	0.05	0.01-0.2	B vs. controls *p* = 0.1
	C	9	0.07	0.03-0.14	C vs. controls *p* = 0.4
	Controls	21	0.06	0.02-0.1	

MMP-2/TIMP-2	A	25	3.2	0.6-5.0	A vs. controls *p* = 0.88
	B	11	3.4	2.8-5.3	B vs. controls *p* = 0.58
	C	9	3.0	1.9-4.8	C vs. controls *p* = 0.09
	Controls	21	3.3	2.0-5.0	

MMP-9/TIMP-2	A	25	0.02	0.01-0.3	A vs. controls *p* = 0.04
	B	11	0.03	0.01-0.3	B vs. controls *p* = 0.04
	C	9	0.1	0.07-0.3	C vs. controls *p* = 0.1
	Controls	21	0.09	0.03-0.28	

**Table 3:** The results of urinary excretions of MMPs and TIMPs in study groups A, B, and C and control groups.

Variable	Group	*N*	Median	Range	Statistical analysis
MMP-1/Cr (pg/mg)	A	25	171.2	89.5-1125.1	A vs. controls *p* = 0.3
	B	11	170.2	93.4-1521.5	B vs. controls *p* = 0.4
	C	9	169.8	47.6-765.4	C vs. controls *p* = 0.4
	Controls	21	164.4	86.9-1488.8	

MMP-2/Cr (pg/mg)	A	25	401.2	120.2-2310.0	A vs. controls *p* = 0.14
	B	11	323.1	87.9-628.3	B vs. controls *p* = 0.6
	C	9	315.8	121.1-399.8	C vs. controls *p* = 0.24
	Controls	21	305.9	129.3-979.3	

MMP-9/Cr (pg/mg)	A	25	1.87 × 10^3^	0.32 × 10^3^ − 7.13 × 10^3^	A vs. controls *p* = 0.002
	B	11	1.01 × 10^3^	0.22 × 10^3^ − 9.36 × 10^3^	B vs. controls *p* = 0.009
	C	9	2.68 × 10^3^	0.56 × 10^3^ − 3.67 × 10^3^	C vs. controls *p* = 0.003
	Controls	21	0.49 × 10^3^	0.14 × 10^3^ − 1.52 × 10^3^	

TIMP-1/Cr (pg/mg)	A	25	7.13 × 10^3^	4.84 × 10^3^ − 17.9 × 10^3^	A vs. controls *p* = 0.04
	B	11	4.98 × 10^3^	1.23 × 10^3^ − 8.77 × 10^3^	B vs. controls *p* = 0.36
	C	9	4.44 × 10^3^	2.56 × 10^3^ − 7.08 × 10^3^	C vs. controls *p* = 0.88
	Controls	21	5.42 × 10^3^	2.05 × 10^3^–13.7 × 10^3^	

TIMP-2/Cr (pg/mg)	A	25	2.98 × 10^3^	0.12 × 10^3^ − 6.69 × 10^3^	A vs. controls *p* = 0.04
	B	11	2.85 × 10^3^	0.34 × 10^3^ − 9.26 × 10^3^	B vs. controls *p* = 0.02
	C	9	2.19 × 10^3^	1.40 × 10^3^ − 10.6 × 10^3^	C vs. controls *p* = 0.01
	Controls	21	0.43 × 10^3^	0.10 × 10^3^ − 2.67 × 10^3^	

MMP-1/TIMP-1	A	25	0.38	0.11-1.0	A vs. controls *p* = 0.2
	B	11	0.32	0.11-0.54	B vs. controls *p* = 0.06
	C	9	0.5	0.30-0.71	C vs. controls *p* = 0.7
	Controls	21	0.44	0.11-0.66	

MMP-2/TIMP-1	A	25	0.1	0.04-1.0	A vs. controls *p* = 0.001
	B	11	0.7	0.11-2.4	B vs. controls *p* = 0.1
	C	9	0.8	0.11-0.92	C vs. controls *p* = 0.2
	Controls	21	0.9	0.20-4.2	

MMP-9/TIMP-1	A	25	1.2	0.03-1.51	A vs. controls *p* = 0.87
	B	11	1.9	0.1-6.4	B vs. controls *p* = 0.7
	C	9	0.9	0.1-1.7	C vs. controls *p* = 0.09
	Controls	21	1.2	0.35-16.2	

MMP-1/TIMP-2	A	25	0.2	0.1-0.3	A vs. controls *p* = 0.8
	B	11	0.12	0.03-0.4	B vs. controls *p* = 0.1
	C	9	0.1	0.03-0.23	C vs. controls *p* = 0.8
	Controls	21	0.1	0.02-0.2	

MMP-2/TIMP-2	A	25	0.05	0.02-0.28	A vs. controls *p* = 0.001
	B	11	0.11	0.01-0.3	B vs. controls *p* = 0.6
	C	9	0.09	0.02-0.41	C vs. controls *p* = 0.1
	Controls	21	0.1	0.02-0.31	

MMP-9/TIMP-2	A	25	0.18	0.06-0.28	A vs. controls *p* = 0.57
	B	11	0.2	0.09-0.28	B vs. controls *p* = 0.7
	C	9	0.09	0.03-0.2	C vs. controls *p* = 0.39
	Controls	21	0.1	0.04-0.32	

Cr: creatinine.

## Data Availability

The data used to support the findings of this study are available from the corresponding author upon request.
